# *In Vivo* Suppression of Autophagy via Lentiviral shRNA Targeting Atg5 Improves Lupus-Like Syndrome

**DOI:** 10.1155/2020/8959726

**Published:** 2020-05-01

**Authors:** Chi-Jui Liu, Shye-Jye Tang, Chun-Che Chou, Guang-Huan Sun, Kuang-Hui Sun

**Affiliations:** ^1^Department of Biotechnology and Laboratory Science in Medicine, National Yang-Ming University, Taipei 112, Taiwan; ^2^Institute of Bioscience and Biotechnology, National Taiwan Ocean University, Keelung 202, Taiwan; ^3^Division of Urology, Department of Surgery, Tri-Service General Hospital and National Defense Medical Center, Taipei 112, Taiwan; ^4^Department of Education and Research, Taipei City Hospital, Taipei 112, Taiwan

## Abstract

In both mouse models and clinical patients with lupus, autophagy levels were significantly elevated and correlated with disease activity. Furthermore, autophagy can promote the survival of B and T cells, plasma cell differentiation, and antibody production. These results suggest that autophagy may promote the progression of lupus by regulating the survival of autoreactive immune cells. Therefore, we aimed at studying whether suppressing autophagy can modulate lupus progression *in vivo*. First, we found that the autophagy levels in splenocytes and lymphocytes of peripheral blood (PB) were elevated and positively correlated with disease severity in lupus-prone mice. The shAtg5-lentivirus, which effectively inhibits autophagy *in vitro*, was then injected into the lupus-prone mice. Autophagy levels in lymph node cells and PB lymphocytes were reduced following Atg5 suppression. We also found that lymphadenopathy and the numbers of plasma cells, CD4^−^CD8^−^, and CD4^+^ T cells decreased in mice treated with the shAtg5-lentivirus. The mice treated with shAtg5-lentivirus exhibited lower levels of proteinuria, serum anti-dsDNA antibody, B-cell activating factor (BAFF), and glomerular immune complex deposition. Therefore, targeting autophagy to moderate overactivated autophagy in immune cells seems to be a novel strategy for combination therapy of lupus.

## 1. Introduction

Systemic lupus erythematosus (SLE) is an autoimmune disease characterized by the production of antinuclear antibodies, such as the anti-dsDNA antibody. In the lupus patients, both the innate and adaptive immune responses are dysregulated [1]. The autoreactive B and T cells may avoid clonal deletion in the primary and secondary lymphoid organs. Autoreactive B cells can be differentiated into plasma cells (PCs) to produce a large number of autoantibodies. These autoantibodies can bind the autoantigens released from apoptotic cells and necrotic cells to form a nucleic acid-containing immune complex (IC) [2]. Innate immune cells like plasmacytoid dendritic cells (pDCs) and myeloid dendritic cells (mDCs) can be activated by these ICs and release various lupus-related cytokines, such as IFN-*α* and B-cell activating factor (BAFF), and proinflammatory cytokines [2–4], which further promote the activation, proliferation, and survival of T and B cells. They are also capable of promoting PC differentiation [3, 5, 6]. Eventually, severe tissue damage and organ failure occur due to the strong inflammatory response brought by the deposition of ICs in various organs, especially the kidney. Therefore, the survival and activation of autoreactive B and T cells are vital in the pathogenesis of lupus. Certain targeting drugs that can deplete B cells or inhibit the activation and survival of B and T cells are currently being developed and used [1, 3, 4, 7].

Macroautophagy (hereafter referred to as autophagy) is a self-eating process that can degrade aged organelles and unfolded proteins. Therefore, autophagy is vital for cell survival under stress conditions, such as nutrient starvation and hypoxia [8, 9]. During the initiation stage of autophagy, autophagy-related gene 13 (Atg13), Atg101-ULK1, and FIP200 proteins can be recruited to form a complex on the phagophore assembly site. Phagophore further recruits other important autophagy-related proteins, such as Beclin-1, Atg5, and LC3, to drive the elongation process. The autophagy elongation complex (*Atg5*-12/16L1) is formed during this stage, and LC3-I is coupled with phosphatidylethanolamine to develop LC3-II. Therefore, the conversion of LC3-I to LC3-II is an important autophagy marker. Once the elongation is completed, the phagophore evolves into autophagosome and subsequently into autolysosome after fusing with lysosome [8, 10]. Previous studies have shown that autophagy was involved in various factors of both innate and adaptive immune responses, including phagosome maturation, pathogen degradation, antigen presentation, clearance of apoptotic debris, and regulation of inflammatory cytokine production [10, 11]. Furthermore, autophagy can deliver viral RNA/DNA to TLR7/9-containing endosome and consequently promote IFN-*α* production in pDCs [12–14]. Studies have also shown autophagy to be essential for T lymphocyte homeostasis, survival, and proliferation [15, 16]. Autophagy has also been demonstrated as important for B cell development in the pre/pro-B stage [17], PC differentiation [18, 19], antibody production by PC [18, 20], and the long-term persistence of memory B cells [21].

In both human lupus patients and lupus-prone mice, significantly elevated levels of autophagy in T cells and B cells have been reported [19, 22]. Autophagy is significantly increased in the bone marrow pre-B and peripheral CD19+ B cells and correlated with disease activity [19]. In addition, autophagy is increased in thymocytes and splenic mature T cells of lupus-prone mice [22]. Arnold et al. found that lupus symptoms, including antinuclear antibody secretion, the number of long-lived PCs, and IgG-IC deposits in the kidneys, decreased significantly in Atg5^f/−^.CD21^cre^×B6.*lpr* mice when compared with control B6.*lpr* mice [23]. Various SNPs in autophagy-related genes are associated with SLE [10]. These results imply that autophagy may promote the survival and proliferation of autoreactive B and T cells and autoantibodies production, thus exacerbating lupus. In the current study, we aim at investigating whether modulating autophagy *in vivo* can improve the symptoms of lupus-prone mice. TREM-1^−/−^.*lpr* mice, which exhibited the disease more aggressively [24] and had a higher autophagy level than B6.*lpr* and wild type mice, were intraperitoneally (*i.p.*) injected with lentiviral-shAtg5. We observed that the *in vivo* suppression of Atg5 can improve the lupus-like disease of TREM-1^−/−^.*lpr* mice and thus might be a novel strategy for combination treatment of lupus patients.

## 2. Materials and Methods

### 2.1. Animals

Mice with three different genotypes were used in the current study, including wild type C57BL/6 (WT), B6.MRL-*Fas^lpr^*/J (B6.*lpr*), and TREM-1^−/−^ (Triggering receptor expressed on myeloid cells-1).*lpr* mice. We purchased B6.*lpr* mice from Jackson Laboratory (Bar Harbor, ME) and generated the TREM-1^−/−^.*lpr* mice in our laboratory [24]. All mice were bred and maintained under specific pathogen-free conditions in National Yang-Ming University's animal center. All mouse experiments were approved by the Institutional Animal Care and Use Committee of National Yang-Ming University.

### 2.2. Kidney Function and Pathology

To determine the proteinuria level, the urine of mice with the indicated genotype was collected and tested using the urine strip at specified time points (Macherey-Nagel). The proteinuria index was as follows: 0 for 0 mg/dL, 1 for 0-30 mg/dL, 2 for 30 mg/dL, 3 for 30-100 mg/dL, 4 for 100 mg/dL, 5 for 100-500 mg/dL, and 6 for values greater than 500 mg/dL.

Mouse kidneys were excised and embedded in the optimum cutting temperature (OCT) compound after being euthanized and were serially cut into 7 *μ*m thick sections. The frozen kidney sections were fixed with precooled acetone (-20°C) at room temperature and then stained with the FITC-conjugated anti-mouse IgG antibody (Jackson ImmunoResearch) and the FITC-conjugated anti-mouse C3 antibody (MP Biomedical). Images were taken at 200× magnification using a fluorescence microscope. We analyzed the fluorescence intensity in each glomerulus using the ImageJ software (at least 25 glomeruli were analyzed per mice). The paraffin-embedded kidney sections were stained with periodic acid-Schiff (PAS) by NTUCM Laboratory Animal Center (Taipei, Taiwan). The 400-fold images were taken, and the percentage and total intensity of the PAS-positive area in the individual glomerulus were analyzed by ImageJ software (at least 20 glomeruli/mouse were analyzed). The PAS staining score was calculated by percentage (0 to 100) × total intensity/100000.

### 2.3. Measurement of Autophagy

Acridine Orange is a cell-permeable green fluorophore for staining the acidic vesicular organelles (AVOs) and evaluating the autophagy quantitatively in individual cells [25] Single-cell suspensions were obtained from murine peripheral blood (PB), lymph nodes, and mouse lung cancer cell line (Lewis lung carcinoma, LLC-1). Cells were stained with 2 *μ*g/mL of Acridine Orange (AO, Sigma-Aldrich) and analyzed using flow cytometry. To evaluate autophagy levels, we calculated the percentage of cells that exhibited red fluorescence (AVOs (%)).

For RT-qPCR, RNA was extracted from the LLC-1 cell using TRIzol reagent (Invitrogen). After reverse transcription, real-time PCR was performed with the ABI StepOnePlus Real-Time PCR System using SYBR green master mix (Applied Biosystems) and primers specific for *Atg5* and *Gapdh*. Atg5 forward: 5′- GCC AAG AGT CAG CTA TTT GAC GTT G-3′; Atg5 reverse: 5′- CTT GGA TGG ACA GTG TAG AAG GTC C-3′; Gapdh forward: 5′-CCT GGA GAA ACC TGC CAA GTA-3′; Gapdh reverse: 5′- GGT CCT CAG TGT AGC CCA AGA-3′.

For western blot analysis, we harvested the protein lysates of murine splenocytes. In the LLC-1 experiments, cells were incubated with 100 nM rapamycin or under a serum starvation condition for 24 hours to induce autophagy prior to protein extraction. Western blot analysis was performed to determine the expression levels of autophagy-related proteins using the following antibodies: Anti-Atg5 and Anti-LC3 antibodies (Novus Biotechnology) and Anti-*β*-actin antibody (Sigma-Aldrich). The Atg5 expression levels and the LC3-II to LC3-I ratio were quantified using the ImageJ software. All values were normalized to *β*-actin and the control group.

### 2.4. In Vivo and In Vitro Knockdown by Lentivirus-Derived shRNA

pLKO.1 plasmid containing shLuc, shAtg5, and lentiviral packing vector, pCMV-deltaR8.91, and pMD.G were purchased from the National RNAi core Facility (Academia Sinica, Taipei, Taiwan). The lentivirus was produced using HEK-293T cells after being cotransfected with pCMV-deltaR8.91, pMD.G, and PLKO.1 vectors and was produced and concentrated by the National RNAi core Facility. For the *in vitro* knockdown, the LLC-1 cells were infected with the lentivirus with 8 *μ*g/mL protamine sulfate for 24 hours followed by puromycin selection for 2 weeks. For the *in vivo* knockdown, 2.5 × 10^8^ R.I.U. shRNA-containing lentivirus was *i.p.* injected twice into TREM-1^−/−^.*lpr* mice at 22 and 30 weeks of age.

### 2.5. ELISA

Mouse total IgG anti-dsDNA antibody (Alpha Diagnostic International) and BAFF (R&D Systems) ELISA were performed in accordance with the manufacturer's instructions. We adopted 150- and 100-fold diluted sera to measure anti-dsDNA and BAFF levels, respectively. Relative anti-dsDNA fold was defined as the ratio between the mouse anti-dsDNA and control 9D7 anti-dsDNA monoclonal antibody.

### 2.6. Flow Cytometry

The superficial cervical, axillary, and inguinal lymph nodes, as well as lymph nodes in the peritoneal cavity, were excised after euthanasia. The single-cell suspension of lymph nodes was obtained by pressing the organs through a 70 *μ*m cell strainer (Corning Life Science). 1 × 10^6^ cells were preincubated with anti-CD16/CD32 antibody for 20 minutes and then stained with antibodies against specific cell surface markers, including CD19, CD3, CD11c, B220, CD21, CD23, CD138, CD4, CD8, and CD25 (eBioscience). For intracellular Foxp3 staining, we incubated cells with fixation/permeabilization solution (BD Biosciences) after surface staining and with anti-Foxp3 antibody (eBioscience). Sample analysis was performed using the BD FACSCalibur.

### 2.7. Statistical Analysis

Nonparametric Mann-Whitney *U* test was used for statistical analysis to compare the two groups. We adopted Pearson's correlation coefficient to measure the association of autophagy levels in PB cells and proteinuria levels. *p* values < 0.05 were considered statistically significant. All statistical analyses were performed using the SPSS software.

## 3. Results

### 3.1. Lupus-Prone Mice Exhibited Increased Autophagy Levels

**Figure 1:**
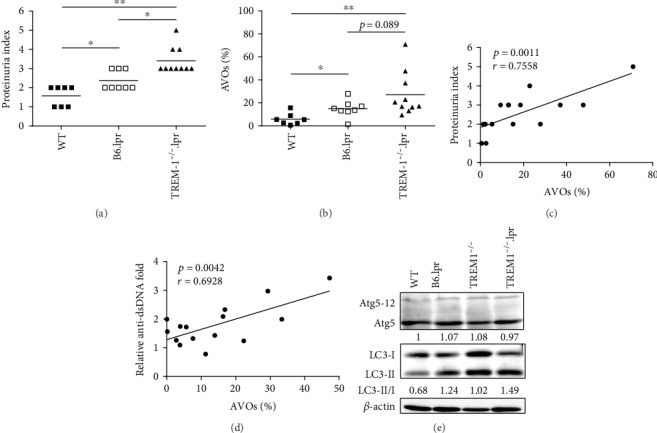
Lupus-prone mice exhibited a higher level of autophagy. (a) The levels of proteinuria in WT, B6.*lpr*, and TREM-1^−/−^.*lpr* mice were measured using urine strips at 32 weeks of age. (b) Peripheral blood cells were harvested and stained with AO, and the autophagy levels (percentage of acidic vesicular organelles (AVOs (%)) in the peripheral blood lymphocytes were then analyzed using flow cytometry. (c) The correlation between autophagy levels in peripheral lymphocytes and proteinuria levels was analyzed. (d) The association between autophagy levels in peripheral lymphocytes and anti-dsDNA levels was analyzed. (e) The spleens of 32-week-old mice were excised and homogenized. Levels of autophagy markers, LC3 and Atg5, were measured with western blot. The expression levels of Atg5 and the LC3-II/I ratio were calculated using ImageJ software, and all values were normalized to the WT control group. Mean values were shown by a bar. ^∗^ represents *p* < 0.05, ^∗∗^ represents *p* < 0.01.

To understand whether autophagy plays a role in lupus, we compared autophagy levels in lupus-prone B6.*lpr* and TREM-1^−/−^.*lpr* mice. TREM-1^−/−^.*lpr* mice showed more severe lupus symptoms than the B6.*lpr* mice. First, we measured the proteinuria levels of WT, B6.*lpr*, and TREM-1^−/−^.*lpr* mice at 32 weeks of age to confirm the development of a lupus-like syndrome. As expected, TREM-1^−/−^.*lpr* mice had the highest proteinuria level, followed by B6.*lpr* and then WT mice (Figure 1(a)). The autophagy levels (acidic vesicular organelles, AVOs %) in the peripheral blood (PB) lymphocytes were then analyzed using flow cytometry after staining with the Acridine Orange (AO) reagent. We found that autophagy was increased in B6.*lpr* mice when compared with WT mice and was higher in TREM-1^−/−^.*lpr* mice (Figure 1(b)). Furthermore, we found that the levels of autophagy (AVOs %) in PB lymphocytes were positively correlated with proteinuria levels (Figure 1(c)). The levels of autophagy in PB lymphocytes were also positively associated with anti-dsDNA levels (Figure 1(d)). We also measured the expression levels of the Atg5 autophagy marker and the LC3-II to LC3-I ratio in the splenocytes. While the expression levels of Atg5 and Atg5/12 complex were similar between the groups, the LC3-II/I ratio was upregulated in the spleen of B6.*lpr* and was higher in the TREM-1^−/−^.*lpr* group (Figure 1(e)). These results imply that autophagy may play a role in the progression of lupus.

### 3.2. Lentiviral-shAtg5 Inhibited the Expression of Atg5 and Autophagy Levels In Vitro

**Figure 2:**
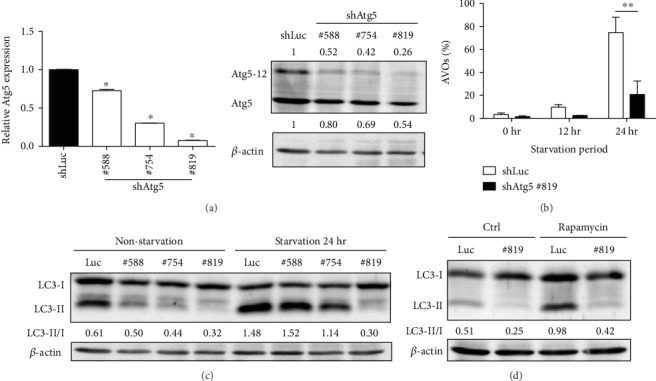
shRNA targeting on Atg5 may efficiently suppress gene expression and autophagy *in vitro*. (a) Mouse lung cancer cell line, LLC-1, was infected with shLuc- or shAtg5-containing lentivirus. Atg5 expression levels were measured by RT-qPCR (Left) and western blot (Right). The numbers show the relative expression of Atg5. (b) Virus-infected LLC-1 cells were starved for 12 and 24 hours. The cells were then stained with AO and analyzed by flow cytometry to determine the autophagy levels (AVOs (%)). The results are representative of three independent experiments. (c, d) The level of LC3 in LLC-1 cells was evaluated using western blot after 24 hours of starvation or Rapamycin treatment (100 nM). The numbers show the relative expression of the LC3-II/I ratio in the individual group. Mean values were shown by a bar. ^∗∗^ represents *p* < 0.01.

To further understand the role of autophagy in lupus, we used lentivirus-mediated shRNA targeting Atg5, an essential protein for phagophore elongation, to suppress autophagy. Prior to the *in vivo* experiment, we confirmed the knockdown efficiency of lentiviral-shAtg5 using LLC-1, a mouse lung cancer cell line. The gene silencing efficiency of three different shAtg5 clones was surveyed using RT-qPCR and western blot. The results suggest that all three clones could effectively suppress the expression of Atg5; of them, clone 819 had the greatest suppressive effect (Figure 2(a), left panel). The protein levels of Atg5 were also confirmed following Atg5 knockdown (Figure 2(a), right panel). To further check the efficiency of Atg5 knockdown in stress-induced autophagy, LLC-1 cells were either incubated in serum-free conditions or treated with rapamycin, a mTOR inhibitor, for 24 hours. The results showed that the levels of autophagy (AVOs %) and the LC3-II/I ratio were significantly increased after starvation (Figures 2(b) and 2(c)) and rapamycin treatment (Figure 2(d)) in the control shLuc group. In contrast, we found dramatic reductions in the autophagy levels and LC3-II/I ratio in the Atg5 knockdown groups, especially in clone 819 (Figures 2(b)–2(d)). According to the above results, clone 819 had the greatest effect on suppressing autophagy and was thus used in the following *in vivo* experiments.

### 3.3. In Vivo Inhibition of Autophagy Improved Symptoms in Lupus-Prone Mice

**Figure 3:**
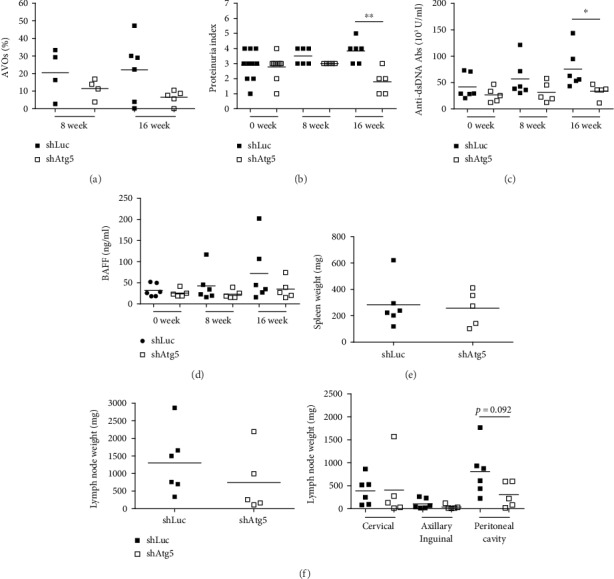
*In vivo* Atg5 silencing attenuated disease development in lupus-prone *Trem-1^−/−^.lpr* mice. shLuc- or shAtg5-containing lentivirus (2.5 × 10^8^ R.I.U) was *i.p.* injected twice into TREM-1^−/−^.*lpr* mice at 22 and 30 weeks old. (a) The autophagy level (AVOs (%)) in peripheral lymphocytes was evaluated using AO staining and flow cytometry. (b) Levels of proteinuria were measured by urine strip at 8 and 16 weeks after the first injection. (c, d) Serum was harvested and anti-dsDNA antibody and BAFF levels were analyzed using ELISA. (e) Mice were euthanized at 38-40 weeks old. Afterward, the spleen and (f) superficial cervical, axillary, and inguinal lymph nodes, as well as lymph nodes in the peritoneal cavity, were excised and weighed. The results are representative of duplicate measurements of each mouse. Mean values were shown by a bar. ^∗^ represents *p* < 0.05.

After confirming the suppressive effect of lentiviral-shAtg5 on autophagy, shAtg5-containing lentivirus was then *i.p.* injected twice into TREM-1^−/−^.*lpr* mice at 22 and 31 weeks of age when these mice exhibited higher autophagy levels and more severe disease. After 8 weeks of virus injection, we measured the autophagy levels in the peripheral lymphocytes and monitored the symptoms of lupus. We found that autophagy levels (AVOs %) in lentiviral-shAtg5-treated mice decreased after the first virus injection and were even lower after the second virus injection (Figure 3(a)). These results suggest that lentiviral-shAtg5 can also suppress autophagy *in vivo*. Even though the suppression of Atg5 did not affect the proteinuria level after the first virus injection, proteinuria was significantly reduced in the Atg5 silencing group after 8 weeks of the second virus injection (Figure 3(b)). We also analyzed the serum levels of anti-dsDNA antibody and B-cell activating factor (BAFF) using ELISA. Compared to the control group, the levels of anti-dsDNA antibody decreased significantly, and BAFF were slightly reduced in lentiviral-shAtg5-treated mice after 8 weeks of the second virus injection (Figures 3(c) and 3(d)).

**Figure 4:**
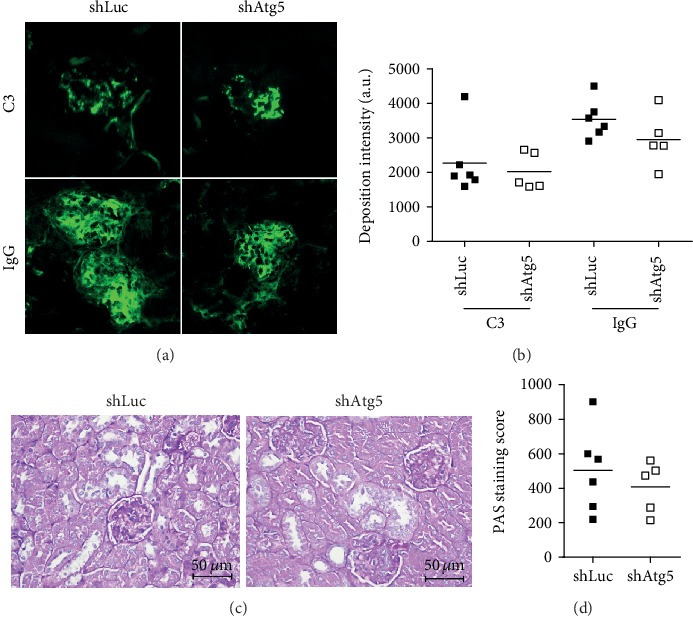
Lentiviral-shAtg5 administrated *Trem-1^−/−^.lpr* mice exhibited improved glomerular immune complex deposition. After shLuc- or shAtg5-containing lentivirus was *i.p.* injected twice into *Trem-1^−/−^.lpr* mice, the mice were euthanized at 38-40 weeks old, and their kidneys were excised. (a) We stained 7 *μ*m thick kidney frozen sections with FITC-conjugated anti-C3 and anti-IgG antibodies. Images were taken at 200× magnification using a fluorescence microscope. (b) The intensity of fluorescence in each glomerulus was quantified using the ImageJ software (at least 25 glomeruli per mouse were analyzed). (c) The paraffin-embedded kidney sections were stained with PAS; the images were taken at 400× magnification. (d) The percentage and total intensity of the PAS-positive area in the individual glomerulus were analyzed by the ImageJ software (at least 20 glomeruli per mouse were analyzed).

When 38 to 40 weeks old, mice were euthanized, and their spleens and lymph nodes were excised and weighed. We found spleen weight to be similar in the control and shAtg5 groups (Figure 3(e)). However, the lymphadenopathy was attenuated in the TREM-1^−/−^.*lpr* mice after suppressing Atg5 expression (Figure 3(f)). Since the deposition of IC is an important characteristic of lupus, IgG-IC and C3-IC levels in the glomeruli were analyzed using immunofluorescence staining. We found that IgG-IC levels were decreased in the glomerulus of the lentiviral-shAtg5 group when compared to those from the control group (Figures 4(a) and 4(b)). In addition, PAS staining scores were lower in the lentiviral-shAtg5 group than in the control group (Figures 4(c) and 4(d)). These results suggest that the inhibition of autophagy *in vivo* could improve a lupus-like syndrome in lupus-prone TREM-1^−/−^.*lpr* mice.

### 3.4. Atg5 Suppression Affected the Immune Cell Composition in the Lymph Nodes

**Figure 5:**
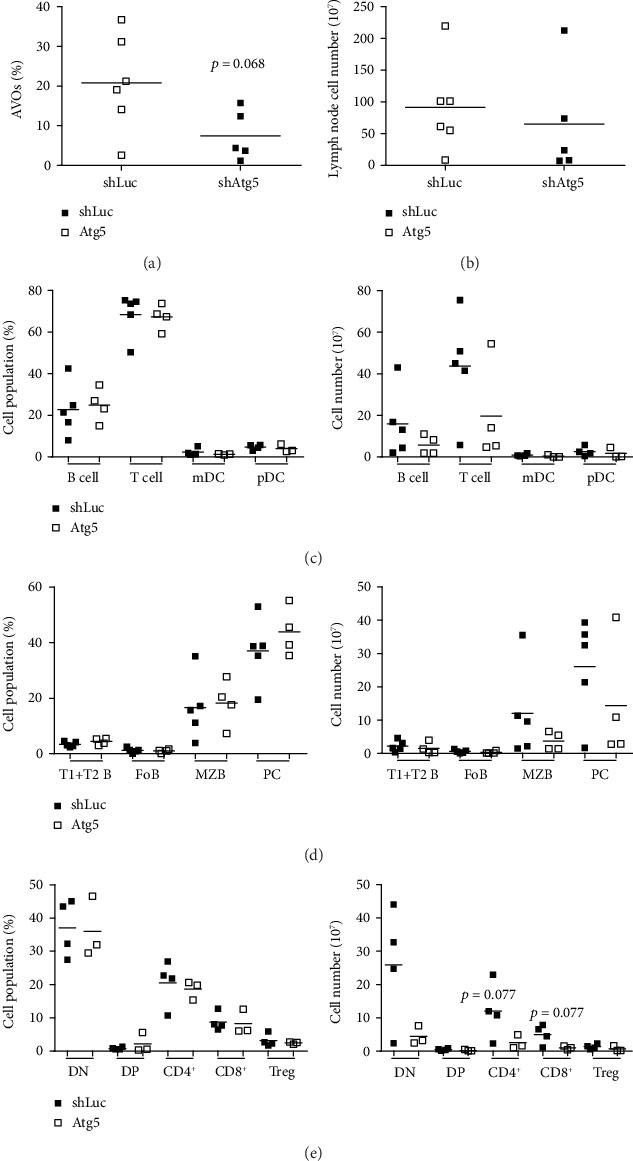
Silencing Atg5 reduced the number of several immune cells in the lymph nodes of *Trem-1^−/−^.lpr* mice. shLuc- or shAtg5-lentivirus-infected mice were euthanized at 38-40 weeks old. Single-cell suspension from the lymph nodes was obtained by passing them through a nylon filter. (a) Single-cell suspension was stained with AO and analyzed using flow cytometry to determine the autophagy level (AVOs (%)). (b) Total lymph node cell numbers were counted and compared. (c, d, e) The percentages (left) and cell numbers (right) of different immune cell subsets were evaluated by flow cytometry using various cell-specific markers, including total B cell (CD19^+^), total T cell (CD3^+^), mDC (CD11c^+^B220^−^), pDC (CD11c^+^B220^+^), T1+T2 B (transitional B cell, CD19^+^CD21^-/low^CD23^-/low^), FoB (follicular B cell, CD19^+^CD21^+^CD23^hi^), MZB (marginal zone B cell, CD19^+^CD21^hi^CD23^+^), PC (plasma cell, CD138^+^), DN T (double-negative T cell, CD3^+^CD4^−^CD8^−^, DP T (double-positive T cell, CD3^+^CD4^+^CD8^+^), T helper cell (CD3^+^CD4^+^), cytotoxic T cell (CD3^+^CD8^+^), and regulatory T cell (CD3^+^CD4^+^CD25^+^FoxP3^+^). Mean values were shown by a bar.

Autophagy levels (AVOs %) declined in the lymph node cells of TREM-1^−/−^.*lpr* mice after the treatment with shAtg5-containing lentivirus (Figure 5(a), Supplementary Figure 1). These results reinforced that lentivirus-mediated shAtg5 can suppress autophagy *in vivo*. As shown in Figure 3(f), the size of the lymph nodes in the lentiviral-shAtg5 group was reduced, and the total cell number of lymph nodes was decreased in lentiviral-shAtg5 treated mice (mean = 65 × 10^7^) when compared with the control group (mean = 90 × 10^7^) (Figure 5(b)). To understand whether the inhibition of autophagy could suppress disease development by affecting immune cell composition, total B, T, mDC, and pDC cells (Figure 5(c)), as well as subpopulations of B (Figure 5(d)) and T (Figure 5(e)) cells in the lymph nodes, were analyzed using flow cytometry. We found that the percentages of immune cell subsets were similar between the control and lentiviral-shAtg5 groups (Figures 5(c)–5(e), left panels). However, the numbers of total B, T cells, marginal zone B cells (MZB), plasma cells (PC), double-negative (DN) T cells, CD4^+^ T cells, and CD8^+^ T cells decreased in the lymph nodes of Atg5 knockdown mice when compared to the control mice (Figures 5(c)–5(e), right panels). Within these immune cells, PC and double-negative T cells were reduced the most in the lentiviral-shAtg5 groups. The above results suggest that suppressing autophagy through lentivirus-derived shAtg5 may hinder the expansion of autoreactive immune cells in the lymph nodes of TREM-1^−/−^.*lpr* mice and may thus inhibit disease development.

## 4. Discussion

Autophagy is closely related to lymphocyte development, activation, polarization, and survival [15–21]. Treg cell-specific deletion of Atg7 or Atg5 resulted in the loss of Treg cells and the development of lymphoid hyperplasia [26]. B cell-specific deletion of Atg5 in lupus-prone mice demonstrated that B cell autophagy is vital for maintaining autoreactive B cells [23]. However, aged (52-week old) mice carrying the myeloid cell-specific knockout of Atg5 (lysozyme M-Cre^+^×Atg5^f/f^) demonstrated lupus-like symptoms, such as increased serum anti-dsDNA antibody, proteinuria levels, and kidney IC deposition, due to the deficiency of LC3-associated phagocytosis, which can promote the clearance of dying cells [27]. Constant levels of autophagy are required to remove unwanted cell contents in all cell types and to clear dying cells in phagocytes [10, 27, 28]. If autophagy is completely inhibited, the autoantigen will be released from the cell contents, resulting in autoimmunity. However, increased autophagy can promote the survival and differentiation of autoreactive B and T cells, as well as antibody secretion [15–20]. Furthermore, hyperactivated autophagy may induce type II programmed cell death and cause autoantigens to accumulate [29]. The above results show that both the hyper- and hypoactivation of autophagy contribute to the pathogenesis of SLE. Therefore, appropriately modulating autophagy seems to be crucial for disease treatment. Nevertheless, since the conditional deletion of Atg5 occurs very early during development, autophagy's role in the later stage of lupus progression remains uncertain. In the current study, we used lentivirus-derived shAtg5 to systemically suppress autophagy and improve lupus-like disease in TREM-1^−/−^.*lpr* mice. These findings suggest that autophagy has a promoting role in the progression of lupus.

BAFF is primarily produced by myeloid cells and is a factor for B cell survival and maturation. Lentiviral Atg5 knockdown in TREM-1^−/−^.*lpr* mice decreased the serum levels of BAFF (Figure 3(d)). Therefore, Atg5 silencing may have both the direct and indirect effects on B cell subpopulations (Figure 5(d)). Interestingly, in addition to PCs, we also found that the number of double-negative T cells, which is one of the major expanded cell types in TREM-1^−/−^.*lpr* mice [24], was dramatically reduced after autophagy suppression (Figure 5(e)). A previous study has shown that double-negative T cells expressed higher autophagy-related proteins, including Atg5, Beclin-1, and LC3, when compared with CD4^+^, CD8^+^, and CD4^+^ CD8^+^ double-positive T cells [15]. Furthermore, Arsov et al. demonstrated that Beclin-1^−/−^ embryonic stem cells could normally differentiate to double-negative T thymocytes after 12 days of culture; however, the double negative T cell population was significantly decreased at day 19, thus, suggesting that autophagy was important for the survival and proliferation of double-negative T cells [30]. Altogether, the autophagy levels of double-negative T cells from TREM-1^−/−^.*lpr* mice may be higher than those from the normal mice and thus may enhance cell survival and proliferation of the autoreactive T cell subpopulation.

Although Atg5 knockdown affected the immune cell composition in the lymph nodes of TREM-1^−/−^.*lpr* mice (Figure 5), we did not find similar effects on immune cell subpopulations in the spleens (data not shown). Intraperitoneal injection of lentiviral-shAtg5 may be the reason because we found a dramatic weight reduction of lymph nodes in the peritoneal cavity (Figure 3(f)). Furthermore, we *i.p*. injected *lentivirus-delivered green fluorescent protein* (GFP) to monitor the target organs through flow cytometry. We found GFP expression in the lymph nodes of the peritoneal cavity and PB lymphocytes and monocytes (Supplementary Figure 2). Therefore, the injection route may affect the target organs and therapeutic efficacy. In summary, we showed in the current study that autophagy was increased in lupus-prone TREM-1^−/−^.*lpr* mice and that the systemic suppression of autophagy using lentiviral-shAtg5 might improve lupus symptoms. Therefore, the use of RNA interference targeting autophagy-related genes to moderate overactivated autophagy in immune cells seems to be a novel strategy for the combination therapy of lupus.

## Data Availability

No data were used to support this study.

## Supplementary Materials

Supplementary Materials Supplementary Figure 1: single-cell suspension from the lymph nodes of shLuc- or shAtg5-lentivirus-infected mice at 38 weeks was stained with AO and analyzed by flow cytometry. (The representative figure of AO stain in Figure 5(a)). Supplementary Figure 2: intraperitoneal injection of lentiviral fluid may circulate to the lymph nodes and peripheral blood. GFP-expressing lentivirus was used to tracing the biodistribution of the inoculated lentivirus in vivo. (A and B) 45-week-old Trem-1-/-.Faslpr mice were injected intraperitoneally with GFP-expressing lentivirus (2.5 × 108 R.I.U., equal units of lentiviral-shLuc was used for the control group). Two days after lentiviral injection, mice were sacrificed and the GFP levels in the lymph nodes, spleen, and mononuclear cells of peripheral blood were analyzed by flow cytometry. (C) 25-week-old Trem-1-/-.Faslpr mice were sacrificed one week after the same injection for flow cytometry analysis as mentioned above. The blue line indicates a lentiviral-shLuc-injected control group while the red line indicates a lentiviral-GFP-injected group. Supplementary Figure 3: single-cell suspension from the lymph nodes of shLucor shAtg5-lentivirus-infected mice at 38-40 weeks was obtained, and the percentages of different immune cell subsets were evaluated by flow cytometry using various cell-specific markers. (The representative figure of immune cell subsets in Figures 5(c)–5(e)).

## References

[1] Rahman A., Isenberg D. A. (2008). Systemic lupus erythematosus. *The New England Journal of Medicine*.

[2] Liu Z., Davidson A. (2012). Taming lupus-a new understanding of pathogenesis is leading to clinical advances. *Nature Medicine*.

[3] Ganguly D., Haak S., Sisirak V., Reizis B. (2013). The role of dendritic cells in autoimmunity. *Nature Reviews Immunology*.

[4] Tsokos G. C., Lo M. S., Reis P. C., Sullivan K. E. (2016). New insights into the immunopathogenesis of systemic lupus erythematosus. *Nature Reviews Rheumatology*.

[5] Vincent F. B., Morand E. F., Schneider P., Mackay F. (2014). The BAFF/APRIL system in SLE pathogenesis. *Nature Reviews Rheumatology*.

[6] Kirou K. A., Gkrouzman E. (2013). Anti-interferon alpha treatment in SLE. *Clinical Immunology*.

[7] Chan V. S., Tsang H. H., Tam R. C., Lu L., Lau C. S. (2013). B-cell-targeted therapies in systemic lupus erythematosus. *Cellular & Molecular Immunology*.

[8] Kaur J., Debnath J. (2015). Autophagy at the crossroads of catabolism and anabolism. *Nature Reviews Molecular Cell Biology*.

[9] Boya P., Reggiori F., Codogno P. (2013). Emerging regulation and functions of autophagy. *Nature Cell Biology*.

[10] Liu X., Qin H., Xu J. (2016). The role of autophagy in the pathogenesis of systemic lupus erythematosus. *International Immunopharmacology*.

[11] Levine B., Mizushima N., Virgin H. W. (2011). Autophagy in immunity and inflammation. *Nature*.

[12] Lee H. K., Lund J. M., Ramanathan B., Mizushima N., Iwasaki A. (2007). Autophagy-dependent viral recognition by plasmacytoid dendritic cells. *Science*.

[13] Zhou D., Kang K. H., Spector S. A. (2012). Production of interferon *α* by human immunodeficiency virus type 1 in human plasmacytoid dendritic cells is dependent on induction of autophagy. *Journal of Infectious Diseases*.

[14] Severa M., Giacomini E., Gafa V. (2013). EBV stimulates TLR- and autophagy-dependent pathways and impairs maturation in plasmacytoid dendritic cells: implications for viral immune escape. *European Journal of Immunology*.

[15] Pua H. H., Dzhagalov I., Chuck M., Mizushima N., He Y. W. (2007). A critical role for the autophagy gene Atg5 in T cell survival and proliferation. *Journal of Experimental Medicine*.

[16] Jia W., He Y. W. (2011). Temporal regulation of intracellular organelle homeostasis in T lymphocytes by autophagy. *Journal of Immunology*.

[17] Miller B. C., Zhao Z., Stephenson L. M. (2008). The autophagy gene ATG5 plays an essential role in B lymphocyte development. *Autophagy*.

[18] Conway K. L., Kuballa P., Khor B. (2013). ATG5 regulates plasma cell differentiation. *Autophagy*.

[19] Clarke A. J., Ellinghaus U., Cortini A. (2015). Autophagy is activated in systemic lupus erythematosus and required for plasmablast development. *Annals of the Rheumatic Diseases*.

[20] Pengo N., Scolari M., Oliva L. (2013). Plasma cells require autophagy for sustainable immunoglobulin production. *Nature Immunology*.

[21] Chen M., Kodali S., Jang A., Kuai L., Wang J. (2015). Requirement for autophagy in the long-term persistence but not initial formation of memory B cells. *Journal of Immunology*.

[22] Gros F., Arnold J., Page N. (2012). Macroautophagy is deregulated in murine and human lupus T lymphocytes. *Autophagy*.

[23] Arnold J., Murera D., Arbogast F., Fauny J. D., Muller S., Gros F. (2016). Autophagy is dispensable for B-cell development but essential for humoral autoimmune responses. *Cell Death & Differentiation*.

[24] Liu C. J., Tsai C. Y., Chiang S. H. (2017). Triggering receptor expressed on myeloid cells-1 (TREM-1) deficiency augments BAFF production to promote lupus progression. *Journal of Autoimmunity*.

[25] Thomé M. P., Filippi-Chiela E. C., Villodre E. S. (2016). Ratiometric analysis of acridine orange staining in the study of acidic organelles and autophagy. *Journal of Cell Science*.

[26] Wei J., Long L., Yang K. (2016). Autophagy enforces functional integrity of regulatory T cells by coupling environmental cues and metabolic homeostasis. *Nature Immunology*.

[27] Martinez J., Cunha L. D., Park S. (2016). Noncanonical autophagy inhibits the autoinflammatory, lupus-like response to dying cells. *Nature*.

[28] He C., Klionsky D. J. (2009). Regulation mechanisms and signaling pathways of autophagy. *Annual Review of Genetics*.

[29] Wang L., Law H. (2015). The role of autophagy in lupus nephritis. *International Journal of Molecular Sciences*.

[30] Arsov I., Adebayo A., Kucerova-Levisohn M. (2011). A role for autophagic protein Beclin 1 early in lymphocyte development. *Journal of Immunology*.

