# Supplementary material for: In Vivo Suppression of Autophagy via Lentiviral shRNA Targeting Atg5 Improves Lupus-Like Syndrome
Source: Biomed Res Int. 2020 May 1;2020:8959726. doi: 10.1155/2020/8959726 (PMC7212279; doi:10.1155/2020/8959726)
Supplement: Supplementary Materials — Supplementary Figure 1: single-cell suspension from the lymph nodes of shLuc- or shAtg5-lentivirus-infected mice at 38 weeks was stained with AO and analyzed by flow cytometry. (The representative figure of AO stain in Figure 5(a)). Supplementary Figure 2: intraperitoneal injection of lentiviral fluid may circulate to the lymph nodes and peripheral blood. GFP-expressing lentivirus was used to tracing the biodistribution of the inoculated lentivirus in vivo. (A and B) 45-week-old Trem-1-/-.Faslpr mice were injected intraperitoneally with GFP-expressing lentivirus (2.5 × 10^8^ R.I.U., equal units of lentiviral-shLuc was used for the control group). Two days after lentiviral injection, mice were sacrificed and the GFP levels in the lymph nodes, spleen, and mononuclear cells of peripheral blood were analyzed by flow cytometry. (C) 25-week-old Trem-1-/-.Faslpr mice were sacrificed one week after the same injection for flow cytometry analysis as mentioned above. The blue line indicates a lentiviral-shLuc-injected control group while the red line indicates a lentiviral-GFP-injected group. Supplementary Figure 3: single-cell suspension from the lymph nodes of shLucor shAtg5-lentivirus-infected mice at 38-40 weeks was obtained, and the percentages of different immune cell subsets were evaluated by flow cytometry using various cell-specific markers. (The representative figure of immune cell subsets in Figures 5(c)–5(e)). [file 8959726.f1.pdf]

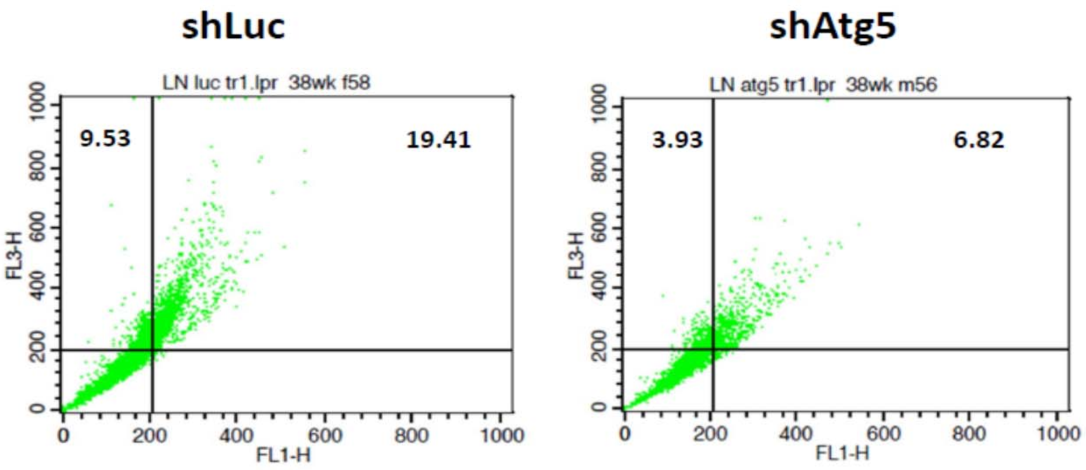

Supplementary Figure 1. Single-cell suspension from the lymph nodes of shLuc- or shAtg5-lentivirus-infected mice at 38 weeks was stained with AO and analyzed by flow cytometry. (The representative figure of AO stain in Figure 5a)

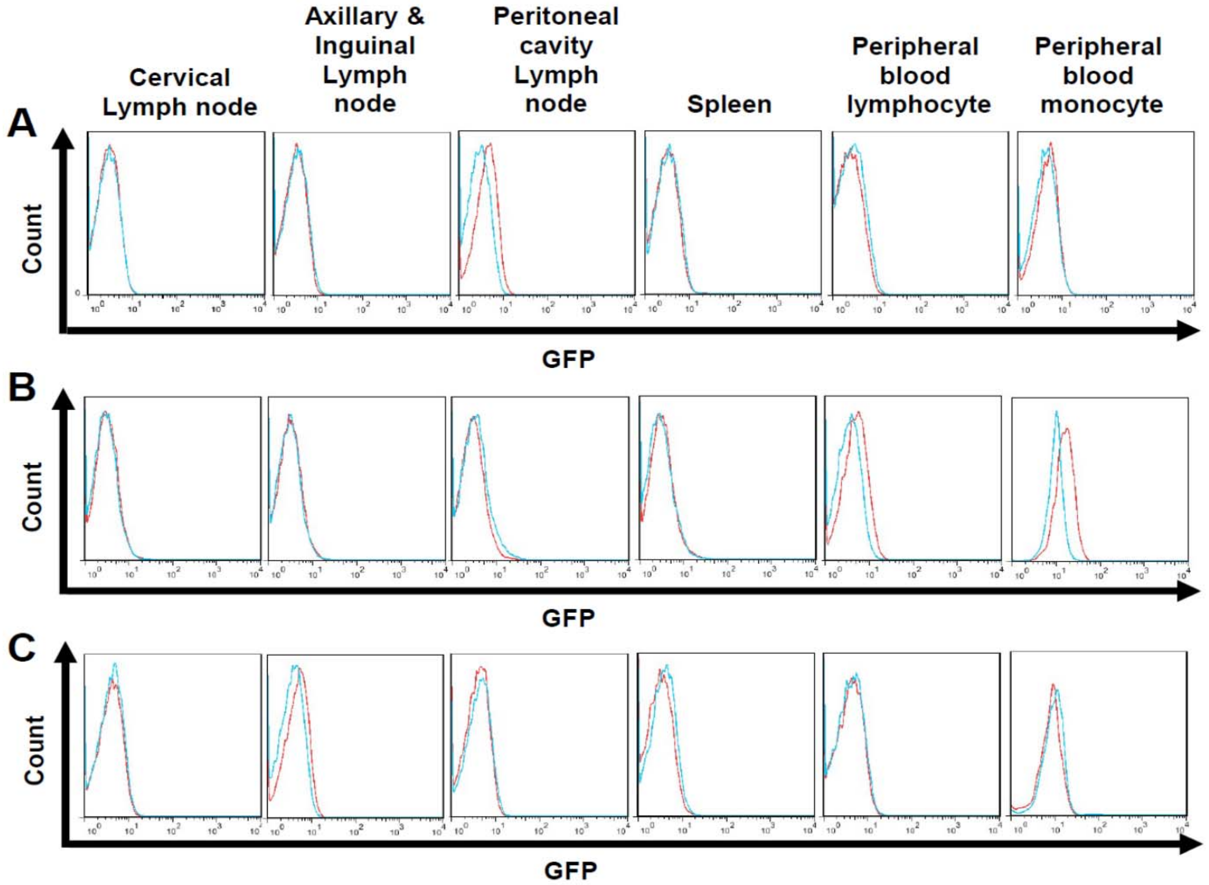

Supplementary Figure 2. Intraperitoneal injection of lentiviral fluid may circulate to the lymph nodes and peripheral blood. GFP-expressing lentivirus were used to tracing the biodistribution of the inoculated lentivirus *in vivo*. **(A and B)** 45-week-old *Trem-1*$^{-/-}$.*Fas*^{lpr} mice were injected intraperitoneally with GFP-expressing lentivirus (2.5x10^8 R.I.U., equal units of lentiviral-shLuc was used for control group). Two days after lentiviral injection, mice were sacrificed and the GFP levels in the lymph nodes, spleen, and mononuclear cells of peripheral blood were analyzed by flow cytometry. **(C)** 25-weeks-old *Trem-1*$^{-/-}$.*Fas*^{lpr} mice were sacrificed one week after the same injection for flow cytometry analysis as mentioned above. The blue line indicates lentiviral-shLuc-injected control group while the red line indicates lentiviral-GFP-injected group.

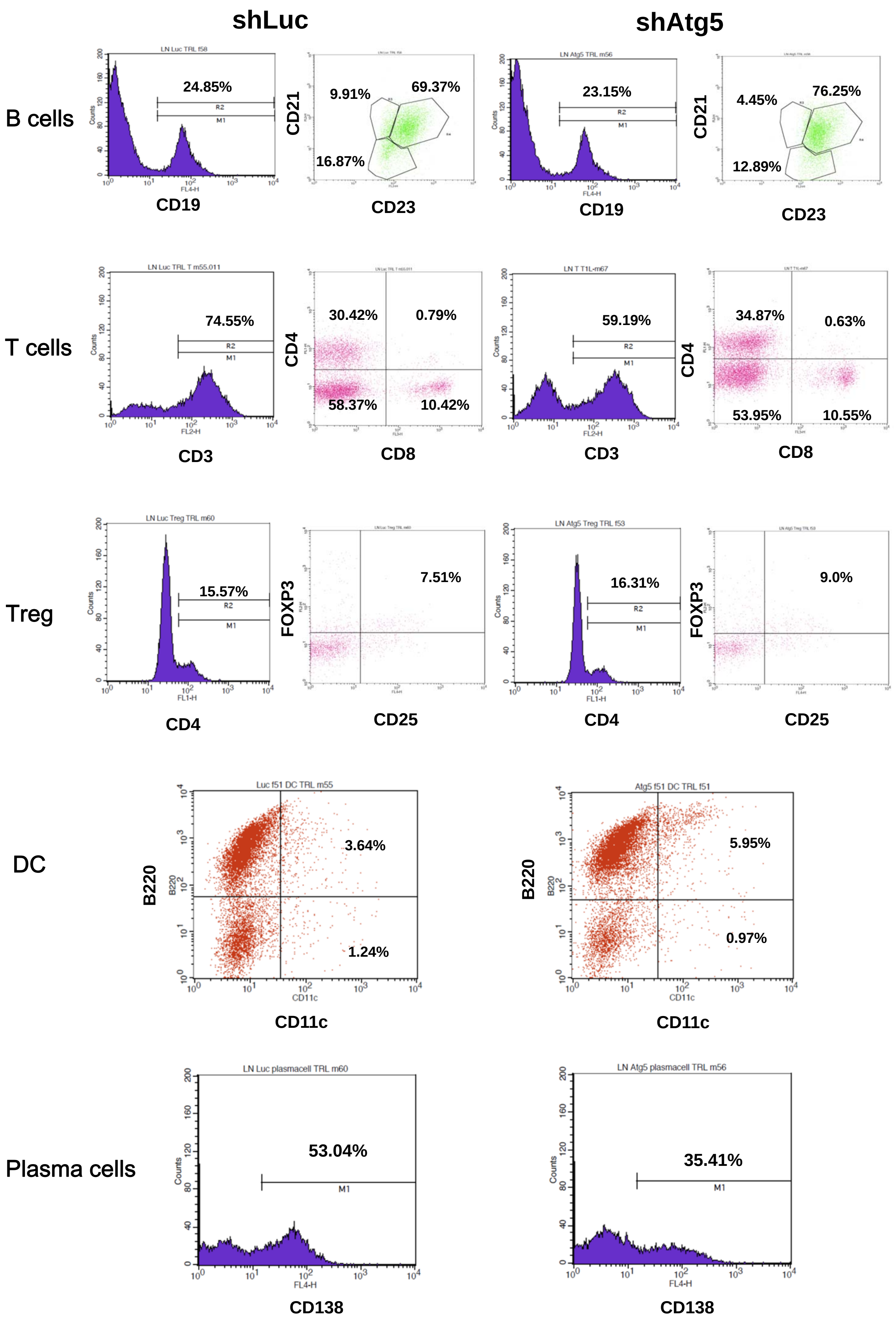

Supplementary Figure 3. Single-cell suspension from the lymph nodes of shLuc- or shAtg5-lentivirus-infected mice at 38-40 weeks was obtained and the percentages of different immune cell subsets were evaluated by flow cytometry using various cell specific markers. (The representative figure of immune cell subsets in Figure 5c,d,e)
